# Severe Acute Respiratory Syndrome Type 2‐Causing Coronavirus: Variants and Preventive Strategies

**DOI:** 10.1002/advs.202104495

**Published:** 2022-01-17

**Authors:** Mehmet Onur Aydogdu, Jennifer L. Rohn, Nazila V. Jafari, Francis Brako, Shervanthi Homer‐Vanniasinkam, Mohan Edirisinghe

**Affiliations:** ^1^ Department of Mechanical Engineering University College London (UCL) Torrington Place London WC1E 7JE UK; ^2^ Department of Renal Medicine Division of Medicine University College London Rowland Hill Street London NW3 2PF UK; ^3^ Medway School of Pharmacy Universities at Medway Chatham ME4 4TB UK

**Keywords:** COVID‐19, prevention, severe acute respiratory syndrome type 2‐causing coronavirus, spike protein mutations, variant of concern

## Abstract

COVID‐19 vaccines have constituted a substantial scientific leap in countering severe acute respiratory syndrome type 2‐causing coronavirus (SARS‐CoV‐2), and worldwide implementation of vaccination programs has significantly contributed to the global pandemic effort by saving many lives. However, the continuous evolution of the SARS‐CoV‐2 viral genome has resulted in different variants with a diverse range of mutations, some with enhanced virulence compared with previous lineages. Such variants are still a great concern as they have the potential to reduce vaccine efficacy and increase the viral transmission rate. This review summarizes the significant variants of SARS‐CoV‐2 encountered to date (December 2021) and discusses a spectrum of possible preventive strategies, with an emphasis on physical and materials science.

## Graphical abstract

Severe acute respiratory syndrome type 2‐causing coronavirus frequently undergoes mutations and modifications of genome expressed on the S region making S protein. Each variant raises global concern as this can cause current treatments to fall behind. Therefore, preventive strategies affecting a broad range of variants are important and are discussed using a physical science and materials science and engineering perspective.

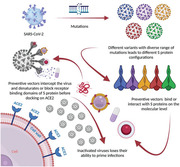

## 1 Introduction

Responsible for the current global state of emergency since December 2019, severe acute respiratory syndrome type 2‐causing coronavirus (SARS‐CoV‐2) is the latest major subspecies of the *Coronaviridae* family and *Betacoronaviruses* (*β*‐CoV) genus.^[^ ^1^ ^]^ This novel virus is extraordinarily successful and dangerous, being the causative agent behind the most significant coronavirus disease to date, the severe respiratory illness coronavirus disease (COVID‐19). After having spread worldwide at a rapid and unbridled pace, SARS‐CoV‐2 was eventually declared a pandemic by World Health Organization (WHO) in March 2020—only a few months after the first laboratory‐confirmed case.^[^ ^2^ ^]^

Coronavirus‐related outbreaks are not a new threat. The first outbreak, the severe acute respiratory syndrome (SARS),^[^ ^3^ ^]^ arose in 2002 and was responsible for more than 8000 infections and 916 deaths in over 29 countries before being quelled by public health measures a few years later.^[^ ^4^ ^]^ The second, Middle East respiratory syndrome (MERS),^[^ ^5^ ^]^ appeared in 2012, is still ongoing but rare, and has spread worldwide with 2279 confirmed cases of which 806 have been fatal.^[^ ^6^ ^]^ Both outbreaks originated from the taxonomic *β*‐CoV genus, which makes them close relatives of SARS‐CoV‐2. However, the current pandemic is substantially more severe compared with any previous coronavirus‐related outbreaks; although SARS‐CoV‐2 is less deadly than its other two cousins, it is far more infectious. Hence, as of 7 December 2021, the number of total cases was 264815815 with 5249793 deaths, highlighting the global impact of the pandemic.^[^ ^7^ ^]^

The general structure of SARS‐CoV‐2 is well‐characterized. The virus contains a linear, single stranded, positive‐sense RNA genome of ≈30K bp that encodes 29 proteins, including four conserved structural proteins: The nucleocapsid protein (N) which protects the genome, and components of the viral envelope including the envelope protein (E), the membrane glycoprotein (M), and the Spike (S) protein. As for all viruses, the complete complement of proteins helps to determine various parameters such as, tissue tropism, attachment, entry, replication, egress, immune stimulation, and ongoing transmission. As such, mutations in any of the genes encoding these proteins can have a profound effect on the host‐pathogen interaction, both positive and negative.^[^ ^8^, ^9^ ^]^ The significant difference in terms of impact, transmission rate, as well as, morbidity and mortality observed during different human coronavirus outbreaks has highlighted how important genomic variation can be. Therefore, understanding the extent and phenotype of coronavirus genomic variation in historic and ongoing strains can provide researchers with the upper hand to counteract future variants.

The S protein has already attracted substantial attention because it plays a crucial role in several vital aspects. The structure of the S protein has been extensively studied because of its importance. It consists of two subunits called S1 and S2. S1 subunit contains the N‐terminal domain and receptor binding domain (RBD), while the S2 subunit harbors the fusion peptide (FP), heptad‐repeat domains (HR1 and HR2), transmembrane domain (TM), and cytoplasmic domain fusion (CT).^[^ ^10^ ^]^ A large membrane glycoprotein that protrudes from the virion, spike mediates the attachment of the virus particle to the host cell receptor angiotensin‐converting enzyme 2 (ACE2) and facilitates the fusion between host and viral membranes which allows entry.

Therefore, spike has a direct influence on viral infectivity. Moreover, S protein presents an attractive antigenic target to the host immune system which is why most vaccine strategies have been aimed at this protein.^[^ ^11^ ^]^ So while mutations occur randomly across the genome, mutations in the S gene in particular have the potential to create variants problematic to their human host both in terms of increased infectivity and immune escape.^[^ ^12^, ^13^ ^]^

**Figure 1:**
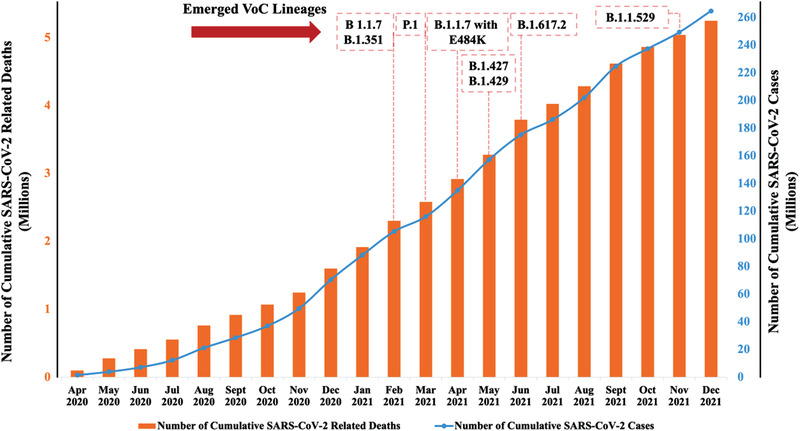
The cumulative number of SARS‐CoV‐2 cases and casualties worldwide. Source: Weekly Epidemiological Updates published by WHO.^[^ ^19^ ^]^

Mutation is recognized as an effective adaptation strategy to increase success, with the risk of deleterious mutations offset by the fitness bestowed by diverse genomes operating in a constantly changing environment.^[^ ^14^ ^]^ RNA viruses in particular tend to undergo more mutations compared with DNA viruses, as they are not amenable to DNA‐based proofreading mechanisms.^[^ ^15^ ^]^ Although SARS‐CoV‐2 is by no means highly mutagenic compared with other RNA viruses, and was originally thought (or at least hoped) to be relatively stable, it has proven to have the ability to produce problematic variants at a high enough rate to constitute a significant threat for human survival and progress in the ongoing pandemic.^[^ ^16^ ^]^ According to the Phylogenetic Assignment of Named Global Outbreak (PANGO) lineages, SARS‐CoV‐2 includes 1542 verified different lineages reported so far.^[^ ^17^ ^]^ Since the pandemic originated from a novel virus that is spreading, mutating, and evolving at a rapid pace, understanding how mutations affect virulence, transmission, and immune evasion is just as important as developing effective treatments and preventive measures. Because we are relying heavily on successful vaccination to control the pandemic, mutations of the S protein have the potential to undermine vaccine efficiency.^[^ ^18^ ^]^ Hence, researchers are looking for alternative ways to inhibit transmission of SARS‐CoV‐2 and its variants. **Figure** **1** shows the cumulative number for SARS‐CoV‐2 cases and casualties mapped in time against the emergence of problematic lineages.

While vaccinations will undoubtedly help control the pandemic, many parts of the world are significantly behind in their immunization programs, in particular the developing world.^[^ ^20^ ^]^ A large amount of activity is also underway to find new therapeutic solutions, and some modest successes have been reported, but thus far a “magic bullet” cure for COVID‐19 has eluded researchers.^[^ ^21^ ^]^ We therefore argue that these herculean biological efforts should be bolstered by complementary research from materials sciences and engineering,^[^ ^22^ ^]^ which include developing surface modifications and polymeric materials that can interact with viruses or act like viruses. Any such approaches should take the structure and characteristics of SARS‐CoV‐2 variants into consideration to achieve broad‐spectrum solutions, including approaches that can be readily adapted in the face of future variants. This review focuses on underlining the relevant characteristics of the currently significant SARS‐CoV‐2 variants while critically appraising the current physical sciences‐based research developments in this vibrant new area.

## 2 Severe Acute Respiratory Syndrome Type 2‐Causing Coronavirus and Variants

As mentioned above, SARS‐CoV‐2 has the potential to create lineages that spread more efficiently and are less responsive to current treatments and preventive measures such as, vaccines.^[^ ^23^ ^]^ Moreover, among all mutations identified within the viral genome of SARS‐CoV‐2, co‐evolutionary analysis of the S gene revealed that it has precisely tuned itself over time to encode a protein with increased affinity to the ACE2 receptor, thereby increasing infectivity.^[^ ^24^ ^]^ Public Health England (PHE) has classified SARS‐CoV‐2 variants into two categories, variants under investigation (VUI) and variants of concern (VoC).^[^ ^25^ ^]^ The United States Centers for Disease Control and Prevention (CDC, United States) has included a third category.^[^ ^26^ ^]^ Specifically, variants can be considered VoC; variants of interest/variants under investigation (VoI or VUI); and variants of high consequence (VoHC). VoI/VUI define variants with mutations affecting receptor binding capability, which can also cause increased transmission rate and lower the effectiveness of the current countermeasures and treatments. On the other hand, VoC is the category of variants that have already made an impact globally in terms of disease severity including some evidence for increased hospitalization, and a potential to undermine current treatments and vaccines. Finally, there is still no SARS‐CoV‐2 variant that falls under the VoHC category, in which the variant would have clear evidence of seriously disrupting current measures and being much more pathogenic.^[^ ^26^ ^]^ However, since SARS‐CoV‐2 is constantly evolving, the status of the variants and their categories are also subject to change, and new variants can also be discovered. To avoid confusion and stigmatization due to different variant names in a rapidly changing environment like this, WHO is also using two main categories for classification purposes (VoC and VoI or VUI), and recently a Greek naming system was introduced to combine all major scientific variant naming systems like PANGO, GISAID, and Nextstrain. Letters of the Greek Alphabet, such as, alpha, beta, and so on are employed to name individual variants, establishing a more coherent approach for public discussions.^[^ ^27^, ^28^ ^]^

### 2.1 Variants of Concern

SARS‐CoV‐2 VoC lineages have already made a noticeable impact on important pandemic parameters, such as increased transmission and reduced vaccine efficiency. A variant named **B.1.1.7** according to PANGO phylogenetic nomenclature (**VOC‐20DEC‐01** as identified by PHE, and **Alpha** according to WHO), was initially acknowledged in November 2020 in the United Kingdom and eventually spread worldwide, manifesting an increased transmission rate of around 50% and increased fatality due to severity of hospitalized cases.^[^ ^29^, ^30^ ^]^ This variant harbored an N501Y mutation within the S protein which increased its ability to bind ACE2 receptors, thereby potentially increasing infectivity;^[^ ^31^ ^]^ Experiments in animal models supported this finding, showing that Alpha had increased cell infectivity compared with the reference genome.^[^ ^32^ ^]^ Moreover, deletions at position 69 and 70 of the S protein led certain diagnostic tests to fail and doubled the infectivity,^[^ ^33^, ^34^ ^]^ and deletion at position 144 was reported to impair binding of certain monoclonal antibodies against the S protein antigen.^[^ ^35^ ^]^ Moreover, a recent preprint reported that Alpha mutations outside the S protein region led to a suppressed host innate immune responses, underscoring the fact that S is not the only region important for immune defense.^[^ ^36^ ^]^

Another significant VoC lineage, **B.1.351** (also known as **VOC‐20DEC‐02** and **Beta**), was initially discovered in South Africa as the most dominant lineages in the same region, and eventually spread internationally. Its rapid spread and aggressive phenotype were reported along with great concern that it might undermine current vaccines.^[^ ^37^ ^]^ Beta contains concerning mutations; E484K substitution in S is one of the most important, which occurs in the receptor‐binding domain and can cause suboptimal immune responses.^[^ ^38^, ^39^ ^]^ Jangra et al.^[^ ^40^ ^]^ recently reported an in vitro microneutralization study where two almost identical viruses (USA‐WA1/2020 virus and a recombinant (r)SARS‐CoV‐2 virus) with a single E484K mutation were selected. The test was conducted using sera taken from participants of different study groups identified as negative, vaccinated, and convalescent with low, moderate, and high levels of Immunoglobulin G (IgG) antibody levels, respectively, based on their SARS‐CoV‐2 S ELISA antibody titer. Results indicated that E484K mutation can affect the binding of serum polyclonal neutralizing antibodies, reducing antibody neutralization, which means that acquired protection from previous infections and vaccination can be evaded.^[^ ^40^ ^]^

Additionally, the K417N mutation in S increases the ability of S proteins to bind to ACE2 receptor, increasing the transmissibility of the variant.^[^ ^41^ ^]^ Moreover, the L18F mutation in S protein was reported to compromise the interaction of neutralizing antibodies which have potential to hinder the effectiveness of vaccines and any relevant antibody‐based therapies.^[^ ^42^ ^]^

Another new VoC, **P.1** (**VOC‐21JAN‐02** and **Gamma**) arose in Brazil and Japan during early January 2021.^[^ ^43^, ^44^ ^]^ Previous reports indicated that the virus accrued various mutations, 12 of which were located within RBDs, including biologically significant mutations in key residues seen in other VoCs, namely, E484K, K417N/T, and N501Y.^[^ ^45^ ^]^ Specifically, in a study investigating molecular characterizations for several current VoCs, E484K, and N501Y mutations resulted in an increase of receptor binding of Gamma S protein to ACE2 receptors—as seen with other VOCs—increasing the ability of the virus to infect host cells, while K417N/T mutation showed a decreased binding affinity—which interestingly is the opposite result to what was reported for the K417N mutation in the context of VoC Beta.^[^ ^46^ ^]^

Another member of the VoC group was declared in April 2021 and named **B.1.617.2**, **VOC‐21APR‐02**, and **Delta**. This variant is a member of sub‐lineage group of a related B.1.617 lineage initially observed in India and contains notable mutations such as L452R, T478K, and P681R, all of which are associated with a possible increase in the transmission rate and enhanced immune escape.^[^ ^47^ ^]^ Consequently, the Delta variant made a substantial impact around the globe. It was also reported that by June 2021, Delta had become the most commonly circulating variant in the UK, constituting a major public health concern since hospitalization rates were relatively higher compared with non‐Delta cases.^[^ ^48^ ^]^ Furthermore, in a recent report summarizing the UK data comparing Delta with Alpha, Delta was shown to be 60% more transmissible. Additionally, people who encounter the Delta variant possess a 17% higher risk of developing COVID‐19 symptoms than those who contract Alpha after the first dose of BNT162b2 or ChAdOx1 vaccines; however, in people who have received two doses, the risk is between 4% and 6%.^[^ ^49^ ^]^

Moreover, it is also important to underline that the B.1.617 lineage is significantly critical because it appears to have the ability to diversify into prominent sub‐lineages with different types of mutations.^[^ ^50^ ^]^ A preprint report presenting a comparison of all sub‐lineages of B.1.617 highlighted their ability to hinder vaccine‐elicited antibodies, implicating L452R and T478K mutations in this phenotype.^[^ ^51^ ^]^ Just as the Delta variant originated from the B.1.617 lineage, the Delta variant in turn spawned various sublineages which were given the alias of AY and investigated under the Delta category.^[^ ^52^ ^]^

A previous member of the VOC category, B.1.1.7 with E484K mutation (no longer a VOC as of June 2021 due to its low profile),^[^ ^53^ ^]^ gained substantial interest because of the problematic E484K mutation seen in Gamma and Beta variants, with lab data underscoring the biological consequences.^[^ ^54^, ^55^ ^]^ Even though other VoCs do not seem to contain the same mutation, recent genome sequence reports revealed that a new variant was derived from current Beta variant and named B.1.1.7 +E484K (formerly as VOC‐21FEB‐02).^[^ ^56^, ^57^ ^]^ This case is also a useful example of currently identified variants that can demonstrate mutations of other verified variants and create a novel VoC. Common mutations presumably reflect convergent evolution on pivotal amino acid residues for key aspects that increase viral fitness. As a case in point, Collier et al.^[^ ^35^ ^]^ reported a study comparing the ability of viruses pseudotyped with wild‐type Beta S proteins, versus controls bearing Beta spike engineered to repair the E484K mutation, to be neutralized by sera from individuals inoculated with the Pfizer‐BioNTech vaccine (BNT162b2). These experiments showed that the E484K substitution was responsible for a substantial decrease in neutralization by both patient antibodies and monoclonal antibodies, which could make the vaccine less effective.^[^ ^35^ ^]^ However, real‐world data amongst vaccinated individuals to determine if VOCs containing this mutation are truly escaping will be important to supplement these in vitro findings.

Finally, a new variant called **B.1.1.529** (**VOC‐ 21NOV‐01** and **Omicron**) emerged in November 2021 and was immediately placed into the VoC category due to its rapidly spreading nature and the fact that it contained a vast number of mutations, including more than 30 changes to the sequence of the S protein.^[^ ^58^ ^]^ Some mutations within the genome of Omicron were the same as those in other VOCs responsible for an increased transmission rate, increased immunoescape properties, and increased chances for catching the disease due to higher affinity to the ACE2 receptors,^[^ ^59^ ^]^ an alarming profile that put the entire world on alert. Moreover, Omicron contained a large number of additions and deletions to the genome whose consequences are as yet unknown.^[^ ^60^ ^]^

Along with the fast‐paced transmission rate of the virus, vaccine effectiveness is one of the main concerns that arose after the case numbers started to increase at a staggering rate. Indeed, initial non‐peer‐reviewed research suggests that Omicron can reinfect individuals with previous exposure^[^ ^61^ ^]^ and even those with two full doses of certain vaccines,^[^ ^62^ ^]^ but much more genetic surveillance and real‐world vaccine efficacy and disease severity data are required to obtain the whole picture about this worrisome new chapter in the COVID‐19 pandemic.^[^ ^63^ ^]^

**Figure 2:**
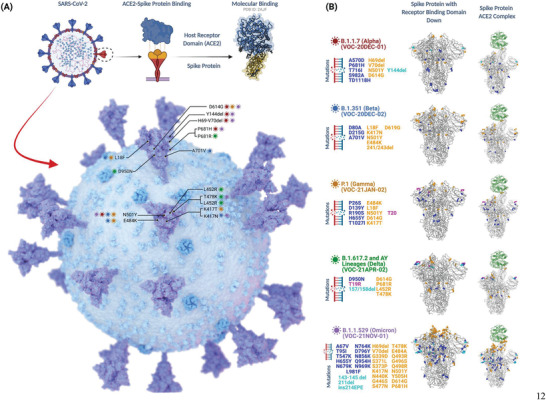
A) Illustration of S protein‐ACE2 binding, and location of the frequently detected mutations within the spike region of SARS‐CoV‐2. B) Detailed visualization of mutations that are found within the genomic structure of current VoC lineages. To display amino acid changes within the S proteins, genomic sequence data of each variant (OU030649.1, OU092214.1, OU061529.1, MZ357460.1, and OL672836.1) was obtained from GenBank database, National Center for Biotechnology Information.^[^ ^66^, ^67^, ^68^, ^69^, ^70^, ^71^ ^]^ Comparative 3D illustrations of the S proteins for each variant were made using CoVsurver, with mutation analysis of hCov‐19 tool provided by GISAID using the hCoV‐19/Wuhan/WIV04/2019 as the reference strain.^[^ ^72^ ^]^ Adapted from “The SARS‐CoV‐2 Variants of Concern,” by BioRender.com (2021). Created with BioRender.com.

**Figure** **2** illustrates the S protein/ACE2 binding interaction structurally and where the key mutations in the major VoCs lie. **Table** **1** summarizes the VoC of SARS‐CoV‐2 currently in circulation and discussed above, including their category, major mutations, key biological effects and number of whole genome sequence submissions made to the Global Initiative on Sharing Avian Influenza Data (GISAID) variant tracking database.^[^ ^64^ ^]^ Even though the database is only a snapshot of variants in circulation, it is an efficient tool to assess the global spread of SARS‐CoV‐2 VoCs compared with the more than 3.2 million genomic sequences submitted in total for SARS‐CoV‐2 between January 2020 and August 2021.^[^ ^65^ ^]^ Finally, since the diversity of the genetic features is observed frequently within the viral genome of SARS‐CoV‐2, it is a vital requirement that genomic surveillance carries on tracking the progress of the variants and identifying ongoing and future mutations. Hence, investigating variants and global infection prevention efforts are crucial to overcome problems of the current pandemic, such as keeping the vaccine efficiency high and effective against different genetic variations, preventing new waves, reducing transmissibility of SARS‐CoV‐2 and its variants, as well as, taking other preventive measures to manage COVID‐19 pandemic.

**Table 1:** Currently recognized Variant of Concern SARS‐CoV‐2 lineages and their important features as at December 2021

Variant names	Origin	Characteristic spike mutations and impact	Other mutations on the spike region	Genetic sequence data submitted to GISAID database		Ref.
				Cumulative	Last 4 weeks	
**B.1.1.7** (**VOC‐20DEC‐01) (Alpha)** ^a)^	United Kingdom	**N501Y**: Enhancing spike/ACE2 binding, **Hv69‐70**: Detection error on specific test kits. **Y144 deletion**: Granting immunoescape properties by compromising alternative monoclonal antibody treatments.	A570D, P681H, D614G, T716I, S982A, D1118H	1148480 submissions from 180 countries	119 submissions from 5 countries	[31, 32, 33, 34, 35]
**B.1.351 VOC‐20DEC‐02 (Beta)** ^a)^	South Africa	**N501Y, L18F, and E484K**: Granting immunoescape properties by compromising neutralizing antibodies, **K417N**: Enhancing spike/ACE2 binding.	D80A, R246I, 241/243deletion D215G,A701V	39420 submissions from 117 countries	3 submissions from 2 countries	[37, 38, 39, 40, 41, 42]
**P.1 VOC‐21JAN‐02 (Gamma)** ^a)^	Japan ex Manaus Brazil	**N501Y and E484K**: Granting immunoescape properties by compromising neutralizing antibodies. Enhancing spike/ACE2 binding.	L18F, H655Y, T20N, T1027I, P26S, D138Y, D614G, R190S, K417N/T	117154 submissions from 91 countries	29 submissions from 4 countries	[43, 44, 45]
**B.1.617.2 and AY lineages VOC‐21APR‐02 (Delta)**	India	**L452R, T478K, and P681R**: Enhancing spike/ACE2 binding, granting immunoescape properties by compromising neutralizing antibodies.	T19R,G142D, del157/158, D950N	3229151 submissions from 183 countries	283703 submissions from 74 countries	[47, 48, 49, 50, 51, 52]
**B.1.1.529 VOC‐ 21NOV‐01 (Omicron)**	South Africa	**N501Y**: Enhancing spike/ACE2 binding **Hv69‐70**: Detection error on specific test kits. **T478K**: Enhancing spike/ACE2 binding, granting immunoescape properties by compromising neutralizing antibodies.	A67V, T95I, T547K, H655Y, N679K, N764K, D796Y, N865K, Q954H, N969K, L981F, 135–145del, 211del, Ins214EPE, G399D, S371L, S373P, K417N, N440K, G446S, S477N, E484A, Q493R, G496S, Q493R, G496S, Q498R, Y505, D614G, P681H	837 submissions from 42 countries	819 submissions from 42 countries	[59, 60]

^a)^ Indicates an exception only for CDC and means variant is a former VoC and moved to another group since September 2021.

### 2.2 Variants under Investigation with Concerning Potential and Variants in Monitoring

VUI, as designated by PHE, and also called VoI by the CDC, is a group of SARS‐CoV‐2 lineages under close surveillance due to their potential to be concerning in the future. As of December 2021, this category is only actively used by PHE, covering a small group of different variants such as **AY.4.2** (**VUI‐21OCT‐01**), **B.1.525** (**VUI‐21FEB‐03 / Eta**) **B.1.617.1** (**VUI‐21APR‐01 / Kappa**), and **B.1.621** (**VUI‐21JUL‐01/Mu**); in contrast, the CDC currently does not classify any variant under this category.

Extremely rapid and ongoing changes occurring within the coronavirus genome comprise different amino acid mutations and as a result, the influence of each variant during the course of the pandemic is also a constantly changing variable. Therefore, the status of the variants also changes depending on the impact of an identified variant on international, national, and regional proportions over time. This third category of VOI is reserved to track variants under surveillance. As of the 21st September 2021, CDC also created a similar category called variants being monitored (VBM) which corresponds to the variants in monitoring (ViM) of PHE. Finally, standardization of terminology has been established and variants with reduced impact in terms of disease severity, transmission rate, and circulation are transferred to that category.

For example, even though the CDC had included **B.1.427** and **B.1.429** lineages (both named **Epsilon**) in the VoC group since the lineage was frequently observed in the United States, and mutations such as, L452R and D614G have been implicated in increased transmissibility and immune escape, PHE has not included this variant in its own VoC list due to its thus far low frequency in the United Kingdom.^[^ ^73^, ^74^ ^]^ As of September 2021, both parties have decided to mark Epsilon lineages under the monitoring category due to the latest situation of the variant.^[^ ^26^, ^75^ ^]^ Currently, most of the variants fall under that category and are kept under observation for preventing any further outbreaks.

**Table** **2** lists currently recognized important SARS‐CoV‐2 lineages outside the VoC group with their defining characteristics. Since there are four VoC, various VUI/VoI, and ViM/VBM currently in circulation and many more predicted to arise, research and current preventive measures should also be tailored to cover differences caused by mutations. For example, knowledge of key variants can guide second‐generation COVID vaccine design. In addition, since the mutations consist of amino acid differences within the S region, including within the ACE2 RBD, using antagonists to prevent binding or otherwise inactivate the virion may constitute novel therapeutic strategies that can be effective regardless of the variant and mutation type.

**Table 2:** Common SARS‐CoV‐2 lineages outside the VoC group and their important characteristics updated as of December 2021

Variant info		Categorization		Origin	Defining S protein mutations	Ref.
Lineage name with WHO classification	Designation	Public Health England	Centers for Disease Control and Prevention			
**P.2 (Zeta)**	**VUI‐21JAN‐01**	VUI	VBM^a)^	Brazil	E484K, D614G, V1176F	[55]
**B.1.427 (Epsilon)**	**N/A**	ViM^a)^, ^b)^	VBM^a)^, ^b)^	United States	L452R, D614G, S13I, W152C	[73, 74]
**B.1.429 (Epsilon)**						
**P.3 (Theta)**	**VUI‐21MAR‐02**	N/A^a)^	N/A	Philippines	E484K, N501Y, P681H, E1092K, V1176F, H1101Y	[76]
**A.23.1 with E484K**	**VUI‐21FEB‐01**	N/A^a)^	N/A	United Kingdom	E484K, R102I, F157L, V367F, Q613H, P681R	[77]
**B.1.525 (Eta)**	**VUI‐21FEB‐03**	VUI	VBM^a)^	United Kingdom	Q52R, A67V, Hv69‐70, F888L Y144 deletion, E484K, D614G, Q667H,	[78]
**B.1.1.318**	**VUI‐21FEB‐04**	VUI	N/A	United Kingdom	E484K, T95I, Y144deletion, P681H, D796H, I82T	[79]
**B.1.617.1 (Kappa)**	**VUI‐21APR‐01**	VUI	VBM^a)^	India	Q1071H, D614G, G142D, E154K, P681R, L452R, E484Q,	[80]
**B.1.617.3**	**VUI‐21APR‐03**	VUI	VBM^a)^	India	L452R, E484Q, P681R, T19R, D950n 156–158 deletion, T478K	[47, 81]
**AV.1**	**VUI‐21MAY‐ 01**	N/A^a)^	N/A	United Kingdom	D80G, T95I, G142D, Y144 deletion, N439K, 5484K, D614G, P681H, I1130V, D1139H	[82]
**C.37 (Lambda)**	**VUI‐21JUN‐ 01**	ViM^a)^	N/A	United Kingdom	75V, T76I, del247/253, L452Q, F490S, D614G, T859N	[83]
**C.36.3**	**VUI‐21MAY‐ 02**	ViM^a)^	N/A	Thailand ex Egypt	S12F, Hv69‐70, W152R, R346S, L452R, D614G, Q677H, A899S	[83]
**B.1.526 (Iota)**	N/A	ViM	VBM^a)^	United States	E484K, S477N, D253G	[84, 85]
**B.1.621 (Mu)**	**VUI‐21JUL‐01**	VUI	VBM	United States	N501Y, E484K, NSP3, T237A, NSP6, T720I, NSP4; T492I, Q160R, NSP12, P323L, NSP13, P419S, T95I.S, R346K, ORF8; T11K, P38S, D614G, P681H, D950N, ORF3a, Q57H, S67F	[75]
**AY.4.2 (Delta)**	**VUI‐21OCT‐01**	VUI^b)^	N/A	United Kingdom	T95I, A222V, Y145H, and other Delta mutations.	[86]

^a)^ Indicates that the threat level of the variant was formerly marked as VUI or VoI and then downgraded to monitoring status (ViM/VBM) or just not available anymore;

^b)^ Indicates the variant formerly marked as VoC.

## 3 Traditional Approaches of the Biological Sciences and Drug Design for Prevention of Severe Acute Respiratory Syndrome Type 2‐Causing Coronavirus and Variants

### 3.1 Vaccine Strategies

Vaccination has always been one of the most essential infectious disease public health strategies, providing protection and prevention against diseases for over 200 years (and even earlier, in the form of smallpox variolation).^[^ ^87^, ^88^ ^]^ Due to the pandemic, an unprecedented global push to create an effective COVID‐19 vaccine in 2020 resulted in development of scores of vaccines, several of which have been approved in record time and rolled out as part of a highly successful worldwide vaccination programs in 2021.^[^ ^89^ ^]^

There are different types of COVID‐19 vaccines currently being developed or administered around the world and each category has a different action mechanism while also being effective on a different level against many distinctive variants. These include 1) virus vaccines, in which the weakened or inactivated virus is administered to promote an immune response; 2) viral vector vaccines, which employ a virus backbone to express coronavirus protein(s), typically Spike; 3) virus‐like particles, which are comprised of “empty” shells without genetic material but sometimes coated with virus proteins to raise an immune response; 4) whole inactivated virus particles; 5) protein‐based subunit vaccines, based on recombinantly produced virus protein(s) (generally S proteins but sometimes and RBD specific); and 6) nucleic acid vaccines.^[^ ^90^, ^91^ ^]^ The latter are one of the most popular, innovative, and successful vaccines, whereby virus‐specific DNA or RNA (protectively coated with various materials) is directly injected into human muscle tissue to guide the cells encoded the viral proteins (typically Spike) to educate the host's immune system on how to recognize the virus. In addition to a full course of vaccine (typically two doses), to date, more affluent countries have employed booster jabs to bolster waning immunity; in the case of Omicron, such boosters seem to be particularly necessary.^[^ ^92^ ^]^ Indeed, some scientists predict that an annual booster might be required to top up population immunity, much as is the case for seasonal influenza.^[^ ^93^ ^]^

According to the WHO, as of 7 December 2021, there were 194 vaccines in pre‐clinical development, while 136 vaccines have already been involved in clinical testing. Among those that made it to the clinical stage, protein subunit‐based vaccines are the most popular (35%).^[^ ^94^ ^]^ Reaching Phase 3 trials, **BNT162b2** from Pfizer/BioNTech and **mRNA‐1273** from Moderna both provide over 90% protection including against different variants. Additionally, vaccines in the viral vector category, each with different efficacy ratios, include ChAdOx1 (**AZD1222)**,developed in a collaboration between AstraZeneca and University of Oxford; **Ad26.COV2‐S**, invented by Johnson & Johnson; **Sputnik V** from Gamaleyab; and **Covaxin** from Bharat Biotech. Moreover, vaccines based on inactivated virus also have reached Phase 3 trials, most notably **CoronaVac,** developed by Sinovac Biotech; and BBIBP‐CorV from Sinopharm. Finally, there are two protein subunit‐based vaccines, which are **NVX‐CoV2373** of Novavax and **EpiVacCorona** industrialized by VECTOR.^[^ ^89^ ^]^

It should also be noted that the efficacy of vaccines has always been tested every time a new variant emerges, and most of the vaccines being used have the capacity to be altered to accommodate escape variants. For example, the rise of dangerous variants including, most recently, Omicron, has stimulated several companies to announce that they were developing and testing “tweaked” vaccines including the mutated antigens.^[^ ^95^ ^]^

There is no doubt that vaccination programs have contributed greatly to control the spread of the virus, reducing the impact of the pandemic, as well as, decreasing hospitalization and death ratio. It is worth noting that the global distribution of the vaccine has been uneven, with more affluent nations being more highly vaccinated than many in the developing world, as is sadly often the case in epidemics^[^ ^96^ ^]^—an unbalanced approach that creates a breeding ground for more dangerous variants that endanger even those countries with good vaccine coverage.^[^ ^97^ ^]^

Given this, although vaccines have clearly been a success, multiple prevention strategies would assist in the pandemic effort. In parallel with vaccine research, vast effort and investment are focused on alternative methods of SARS‐COV‐2 prevention such as, repurposed and traditional drug approaches and physical/materials science approaches.

### 3.2 Traditional and Repurposed Antivirals and Drugs

Using antiviral medicines against SARS‐CoV‐2 is a promising approach for eliminating their ability to infect host cells. Remdesivir is one of the most popular antiviral agents found to be effective against SARS‐CoV‐2, which also gained Food and Drug Administration (FDA) approval as an investigational therapeutic for this purpose. The drug is known to block RNA replication of SARS‐CoV‐2. Even after virus entry into cells, administration of remdesivir can effectively protect the patient and prevent infection.^[^ ^98^, ^99^ ^]^ Remdesivir plays a significant role in protection and was reported to show a synergistic effect with another antiviral drug in in vitro tests. Choy et al.^[^ ^100^ ^]^ reported that using remdesivir on its own was able to inhibit SARS‐CoV‐2 replication with EC_50_ at 23.15 µM. The study also suggested that combination therapy can be more beneficial in terms of clinical benefits; the synergistic combination of remdesivir at 6.25 with 0.195 µM emetine, another antiviral agent could inhibit the viral yield up to 64.9%.^[^ ^100^ ^]^ Even though the results were promising, WHO is still advising against administering remdesivir in addition to usual care due to several reasons such as, low certainty of the evidence, uncertain substantial variability, and not being thoroughly investigated.^[^ ^101^ ^]^ Remdesivir is also categorized as a highly expensive antiviral drug which is also hard to find because of the limited stock.^[^ ^102^ ^]^

Therefore, despite promising results obtained from remdesivir administration at different stages of COVID‐19, the drug is still far from being a game‐changer in this field, while more research and new data are vitally required to assess pros and cons.

Different antiviral drugs already designed against different pathogens (Arbidol, Baloxavir, Laninamivir, Oseltamivir, Peramivir, Zanamivir) were tested for their efficacy against SARS‐CoV‐2. These experiments revealed that Arbidol (Umifenovir), an antiviral medicine already used for influenza treatment, was significantly more promising for blocking cell entry, with the added advantage of eliciting anti‐inflammatory activity.^[^ ^103^ ^]^ Arbidol was further studied at the molecular and structural level in terms of S protein interactions, and the regions where the drug binds S were identified. Since the membrane of the SARS‐CoV‐2 and S protein are required for cell adhesion through ACE2 receptors, and trimerization is an obligatory step for the fusion of the virus to the host, arbidol was proposed to block these interactions thereby rendering the virus less infective. Subsequently, arbidol was reported to have successfully suppressed trimerization of the SARS‐CoV‐2 S glycoproteins and resulted in “immature” or “naked” virion particles, which compromised their ability to infect cells.^[^ ^104^ ^]^

Another repurposed drug currently that is currently being evaluated against SARS‐CoV‐2 is a glucocorticoid, namely dexamethasone (DEX), which is an immune modulator actively investigated and used over the years.^[^ ^105^ ^]^

Zhang et al.^[^ ^106^ ^]^ reported in a study using pseudoviruses equipped with S that DEX was capable of harboring on ACE2 receptors if applied before the viral invasion. Thus, blocking the virus entry into cells by harboring on ACE2 receptors. In terms of binding affinity, results indicated that the concentration of antibody (*K*_D_) value measured amongst DEX and ACE2 was (9.03 ± 0.78) e‐6 M, which was reported to be higher than the *K*_D_ value between S and ACE2, confirming the binding affinity favors the S protein instead of DEX. Even though DEX was not a competitive factor in the race for binding to ACE2, authors reported that having DEX administrated before the virus invasion provides sufficient attachment to ACE2 that can act as a preventive measure for a possible viral invasion.^[^ ^106^ ^]^ Administration of DEX also found a place in controlled clinical trials. The drug was administered to patients on respiratory support (mechanical ventilation or oxygenation) to ameliorate lung injuries related to inflammation. Results indicated that 28‐day mortality rate was reduced with DEX usage in patients on respiratory support.^[^ ^107^ ^]^ Even though the whole glucocorticoid therapy was challenged due to cited lack of evidence of improved clinical conditions and delayed viral clearance in the past, current procedures shows that DEX already found itself a reliable place in the battle against COVID‐19, used as a frontline treatment in the UK hospitals and globally, which decreases the mortality rate in patients at severe stages of COVID‐19.^[^ ^108^, ^109^, ^110^ ^]^

More recently, repurposed oral antivirals against COVID have come to the fore. The oral route is the most convenient, allowing use in the community, which can expand the utility of COVID treatments. In this context, MERCK's Molnupiravir, which has already granted emergency use authorization, and Paxlovid from Pfizer, are good examples. It is reported that Molnupiravir, originally developed to fight influenza, could reduce the risk of hospital admission or death by 50% in mild and moderate cases regardless of the variant type of the virus when administered up to 5 days after the beginning of the symptomatic phase, even when the patient carries a risk factor.^[^ ^111^ ^]^ The drug works in a unique way, in which the replicating viral genome is heavily mutagenized by the drug's metabolites over the five‐day treatment, until the mutational burden cripples the virus permanently.^[^ ^112^ ^]^ However, concerns have been raised since the host DNA may also be at risk of mutations; indeed, a previous study suggested that Molnupiravir can mutate mammalian DNA as well.^[^ ^113^ ^]^ Despite this, the FDA has approved the drug due to its success but also expressed concerns about the side effects which should be carefully evaluated.^[^ ^114^ ^]^

On the other hand, Pfizer reported that Paxlovid is 89% effective against COVID‐19 when administered within 3 days of the beginning of the symptomatic phase.^[^ ^115^ ^]^ Paxlovid is a main viral protease inhibitor drug based on disrupting the protein synthesis mechanism of the virus, inhibiting its ability to replicate. It was developed by combining an antiviral called PF‐07321332, a variant of a drug originally used to treat feline infectious peritonitis, and ritonavir which was a repurposed drug originally developed against HIV.^[^ ^116^ ^]^ In detail, as explained by the company, PF‐07321332 is engineered to block the activity of SARS‐CoV‐2‐3CL protease (coronavirus 3‐chymotrypsin‐like‐protease), which is a key replication enzyme for coronavirus, while the ritonavir reduces the metabolism rate of PF‐07321332 in human body, allowing it to remain active for a prolonged period.^[^ ^117^ ^]^ Its Phase 3 trial will be finished in early 2022 and the FDA is currently considering it for emergency use authorization.

Unfortunately, not every attempt for repurposing drugs against SARS‐CoV‐2 resulted in successful outcomes despite the public attention created when they were initially introduced. Originally used as malaria drugs, chloroquine, and hydroxychloroquine were two other drugs trialed against SARS‐CoV‐2. Even though there was considerable evidence documented for hydroxychloroquine showing effective and promising in vitro results in terms of antiviral activity, long‐term toxic effects plus clinical trials showing no decrease in mortality have reduced the credibility of hydroxychloroquine.^[^ ^118^ ^]^

### 3.3 Antibiotics

Antibiotics have also been studied as potential SARS‐CoV‐2 therapies, despite their specificity against bacterial infection. A preprint article of Yu et al.^[^ ^119^ ^]^ explored Teicoplanin, a glycopeptide‐based antibiotic discovered in 1978 and regularly used against bacterial infections due to its low toxicity. This drug was of particular interest because it inhibits cathepsin L, an enzyme compulsory for viral cell entry mechanisms for several viruses including SARS‐CoV and MERS‐CoV. Earlier work by the same author reported that cathepsin L cleavage sites were present on the S protein of SARS‐CoV‐2. In the subsequent paper, pseudoviruses were employed to confirm that Teicoplanin can target these sites and alter virus‐cell attachment. Moreover, the study suggests that even in a situation where S/ACE2 binding was inevitable, the drug could still inhibit subsequent entry events. Using teicoplanin is reported to prevent infection by blocking S protein and preventing the virus binding on the cysteine protease cathepsin L enzyme.^[^ ^119^ ^]^

In addition, a glycopeptide antibiotic called dalbavancin was predicted by a virtual screen to be effective against SARS‐COV‐2. Wang et al.^[^ ^120^ ^]^ published a comprehensive study investigating FDA‐approved peptide drug libraries, looking particularly at ones that can bind to four key amino acid residues in spike that mediate ACE2 binding; dalbavancin was one of these candidates. In lab experiments, dalbavancin bound to ACE2 with extraordinary affinity and prevented interactions with S protein, reducing the infectivity of the virus. Moreover, viral replication was also shown to be inhibited in ACE2‐rich Vero E6 cells with an EC50 of approximately 12 nm.^[^ ^120^ ^]^

### 3.4 Antibody Therapy

Antibody therapy has been studied as an additional way to provide protection against SARS‐CoV‐2, preventing hospitalization and death by intercepting the virus and neutralizing it before the virus binds to cells.^[^ ^121^ ^]^ There are different approaches, and various attempts reported using naturalizing antibodies and nanobodies for prevention and treatment of COVID‐19.^[^ ^22^ ^]^ Several strategies have gained emergency authorization or are in clinical trials at various stages.^[^ ^122^ ^]^ Although their effectiveness is not assured against viruses with future mutations, recent studies suggest that monoclonal antibodies designed to mimic the immune response can be effective against VoC.^[^ ^123^ ^]^ Based on data showing that mortality rate might be related to high viral loads, Weinreich et al.^[^ ^124^ ^]^ reported interim results in an ongoing clinical trial of COVID‐19 patients. Patients were administered with REGN‐COV2, an antibody cocktail comprised of two human IgG1 antibodies targeting the RBD of S protein; results showed reduced viral load without showing side effects that can outweigh the benefits. Effects on clinical outcome have yet to be determined.^[^ ^124^ ^]^

On the other hand, using convalescent plasma has also been attempted due to its success in historical epidemics including previous coronavirus outbreaks. The technique involves collecting plasma containing antibodies from individuals after infection instead of producing antibodies in the laboratory.^[^ ^125^ ^]^ It is reported that receiving convalescent plasma within the three days of COVID‐19 diagnosis would be the most effective way which could reduce the mortality rate compared to individuals who have received the treatment after three days, or no treatment at all.^[^ ^126^ ^]^ However, despite the fact that specific studies reported promising and successful results, when the topic is taken into account, convalescent plasma therapies are more like something that needs to be assessed carefully while pros and cons should be weighted meticulously in order to decide if it could be beneficial according to the condition of the patient and course of the disease since the majority of the randomized trials in this field have reported failing to show progress and improvement in terms of clinical outcomes.^[^ ^127^ ^]^

### 3.5 Peptides and Proteins

Peptides and peptide‐based preventive approaches also have the potential to be exploited for antiviral strategies, in particular the use of natural or synthesized peptides to block ACE receptor binding. Xia et al.^[^ ^128^ ^]^ reported a pan‐CoV fusion inhibitor designed to inhibit S protein bindings for five different coronavirus types (including, SARS‐CoV and MERS‐CoV). A peptide with 36 residues called EK1 was found to work by blocking HR1 domains of the S protein.^[^ ^128^ ^]^ Having obtained successful results, they focused on SARS‐CoV‐2 in a follow‐up study. In this paper, investigation of the crystal structure of both HR1 and HR2 domains on the S protein led researchers to understand that the binding capacity of SARS‐CoV‐2, as measured by membrane fusion, is significantly higher than that of related coronaviruses due to several mutations, and the increased binding capacity can be manipulated using peptides to prevent SARS‐CoV‐2 S‐mediated membrane fusion. Therefore, researchers coupled the previously discovered EK1 peptide with cholesterol molecules and created lipopeptides with varying amino acid sequences. Among all, EK1C4 was found to be the most successful. The half maximal inhibitory concentration (IC_50_) for S protein‐induced membrane affinity was 1.3 nanomolar (nm), and for pseudovirus infection it was 5.8 nm; both are significantly more effective and more promising compared with results obtained from EK1 and other lipopeptides that were tested.^[^ ^129^ ^]^ Furthermore, Karoyan et al.^[^ ^130^ ^]^ published a study on about 12 different peptides designed to mimic the N‐terminal helix of the ACE2, which possess the majority of the important binding residues for S protein, with the aim of hindering the interactions between them. First, the authors compared manufactured peptides against SARS‐CoV‐2 isolated from a symptomatic host to test their ability to inhibit viral replication. Two peptides were found to be superior, each sharing a homotyrosine residue, and one was selected and used as a basis to design two additional peptides. These three peptides were compared in terms of cytotoxicity and S protein affinity strength, which also means their ability to inhibit ACE2‐S binding by blocking the S region. Moreover, cytotoxicity and their ability to prevent viral infections in a dose‐dependent manner were tested. Median inhibitory concentration was calculated between 42 and 53 nm for the three peptides after they were bound to the S protein with strong affinity, which was promising. Results also indicated that no toxicity was observed even though concentrations tested were up to 150 times higher than the IC_50_. Therefore, these peptides have the potential to be an effective antiviral tool, but more research is needed.^[^ ^130^ ^]^ Computational approaches are also being taken to design novel inhibitors of the S/ACE2 interactions. Cao et al.^[^ ^131^ ^]^ reported a two‐sided de novo design approach aimed at creating mini‐proteins, known as “minibinders,” that would bind to S protein and prevent ACE2 interactions. Scaffold structures were computer‐designed to encompass the ACE2 region known to interact with S protein, and also regions in the RBDs. 10 designs could bind the S RBD, with IC_50_ ranging between 24 pm and 35 nm and confirmed by cryo‐electron microscopy, and this may one day serve as the basis for a new therapeutic strategy.^[^ ^131^ ^]^

## 4 Physical Science Approach for Prevention of Severe Acute Respiratory Syndrome Type 2‐Causing Coronavirus and Variants

As discussed in previous sections, the SARS‐CoV‐2 genome is constantly evolving and as a result, the ability of glycoprotein surfaces of the S protein to bind to the ACE2 receptor of host cells can alter the viral infectivity. This binding affinity might be manipulated to control the spread of the virus in a broad range of applications. Therefore, it is a crucial requirement to understand the surface properties of the virus particle and develop surfaces that might attract or repel the virus, depending on the application type. However, current biophysical and biochemical studies cannot keep pace with the rapid evolution of mutations, and comprehensive investigation of the surface properties of each single variant is not practical. It is worth noting that altered amino acid composition does not necessarily lead to altered surface characteristics.

It was reported by Hatmal et al. that the certain mutations (Leu to Phe486, Pro to Ala475, Pro to Glu484) found in the S region of SARS‐CoV‐2 grant the virus more ordered structure and hydrophobic features compared with previous coronaviruses.^[^ ^132^ ^]^ This study is an interesting starting point, but the next step is to investigate the surface characteristics of key variants. Doing so may allow the development of tools to lower viral transmission by controlling the inanimate surface‐virus interactions, while viral infectivity can be tackled by preventing S protein from binding to ACE2 receptors of cells.^[^ ^76^, ^133^ ^]^

Even though variant‐specific data is limited, the SARS‐CoV‐2 reference strain (wild type) itself is extensively studied in terms of surface interactions. Aydogdu et al. described surface persistence of the virus on inanimate surfaces, from hours to days, depending on the surface material type and other factors which may influence viral transmission.^[^ ^9^ ^]^ These results support the initial idea of creating virus‐binding and virus‐repelling surfaces which can be tailored given our understanding of specific surface mechanisms that can be controlled by certain physical forces. Studies have also shown that van der Waals forces, electrostatic interactions, and positive zeta potential are the main factors that influence the virus binding to a surface, as SARS‐CoV‐2 is reported to carry a positively‐charged capsid. Therefore, negatively‐charged surfaces might induce virus attachment. Moreover, pH, pI, and iconic strength can also manipulate the ability of the virus to persist on surfaces along with porosity and surface roughness.^[^ ^134^, ^135^, ^136^ ^]^ It is helpful to consider these physio‐chemical interactions at two different points, namely, before and after viral entry into the body. Before transmission happens, virus‐repelling surfaces can be used to create inanimate objects that can prevent virus particles from docking and persisting, possibly also including dual‐purpose materials with virostatic or virocidal properties. This kind of approach can stop inanimate surfaces from becoming fomites that act as indirect viral transmission vectors. In the second category, because direct virus transmission is the primary cause of most new cases,^[^ ^137^ ^]^ using previously explained physicochemical properties of the virus and S protein binding affinity may potentially be exploited to design drugs tailored to slow or inhibit viral replication even after initial infection, which could reduce the viral load and concomitant disease severity.^[^ ^138^ ^]^ Coronavirus and surfaces is a key theme in the fight against COVID‐19 and much information on this topic is already being compiled.^[^ ^139^ ^]^

### 4.1 Novel Materials at the Molecular Level

Given that the VoC group harbors mutations that increase virus attachment to ACE2,^[^ ^31^, ^41^, ^46^ ^]^ and that coronavirus lineages seem to have a higher affinity for docking on ACE2 receptors, creating and deploying decoy targets as a therapy might reduce SARS‐CoV‐2 infectivity; such decoys could be readily adopted to account for any future variants. For example, Wang et al.^[^ ^140^ ^]^ studied human embryonic kidney (HEK)‐293T cells, known to highly express ACE2 receptors, with membrane‐based, ACE2‐rich nanoparticles against SARS‐CoV‐2; in this study, viral S proteins selectively targeted the nanoparticles and neutralized the ability of S to bind ACE2, leaving the host kidney cells unscathed.^[^ ^140^ ^]^ Furthermore, Rao et al.^[^ ^141^ ^]^ reported “nano decoys” to achieve a similar effect involving a versatile two‐step approach to neutralize SARS‐CoV‐2 and related immune responses. These nanodecoys consisted of cellular membrane nanovesicles originated from human monocytes combined with genetically engineered cells abundant in ACE2 and cytokine receptors. Virus entry into cells was prevented, as the decoys provided a higher binding affinity for S proteins; essentially, the virus particles were intercepted by the decoy's ACE2 regions. Moreover, in a mouse model of acute pneumonia, the decoys eliminated disease symptoms and lung injury via blocking harmful cytokine expression.^[^ ^141^ ^]^ In another study, Li et al.^[^ ^142^ ^]^ synthesized ACE2 nanodecoys using human lung spheroid cells, again with the strategy of neutralizing SARS‐CoV‐2 by attracting the RBDs of S protein. In this study, the delivery method was designed to be inhalation instead of traditional methods, which would situate the decoys in the main site of initial infection. Nanodecoys were tested against S protein and pseudoviruses synthesized to mimic SARS‐CoV‐2. The results verified that nanodecoys had a high affinity for the S proteins and neutralized viral infection. Moreover, animal tests showed that nanodecoys were found to be effective even after 72 h after administration in the lungs, providing excellent protection against the virus without causing host toxicity.^[^ ^142^ ^]^

Polymeric nanoparticles can also offer new opportunities for disrupting interactions between the S binding domain and ACE2 receptors. The relevant polymers also benefit from being well‐proven candidates for pharmaceutical and biomedical applications in various fields, being already approved for use in patients.^[^ ^143^ ^]^ Zhang et al.^[^ ^144^ ^]^ described neutralization of SARS‐CoV‐2 virus with nanosponge structures consisting of a polymeric core made of poly(lactic‐*co*‐glycolic acid) coated with either of two different cell membranes extracted from lung epithelial cells and macrophages—two cell types that have a higher chance of encountering SARS‐CoV‐2 in natural infections. The main idea was to create a structure displaying the elements of the original target of the virus, including ACE2 receptors, and nanosponges of both cell types were found successfully to decrease infectivity via binding and inactivation.^[^ ^144^ ^]^

Additionally, a study published by Milewska et al.^[^ ^145^ ^]^ documented the effectiveness of the HTCC polymer (catatonically modified chitosan N‐(2‐hydroxypropyl)‐3‐trimethylammonium chitosan chloride); a material that is not yet approved for usage in humans, while its toxicity is controversial and being discussed. Interestingly, its spectrum of action was not limited to SARS‐CoV‐2, but also worked against MERS‐CoV, implying that such a strategy might be beneficial for different variants and coronavirus types in future.^[^ ^145^ ^]^ Moreover, within the scope of the Milewska et al. study, in additional work published by the same author, HTCC has emerged as a promising material to fight against members of the *Coronaviridae* family. The material was found to be effective at interacting with S proteins and blocking the viral entry. Furthermore, HTCC obstructed viral replication in human airway epithelium.^[^ ^145^, ^146^ ^]^

Inorganic polyphosphate has also been shown to block S protein. For example, Neufurth et al.^[^ ^147^ ^]^ reported using inorganic polyphosphate (polyP), which is an active and morphogenetic polymer known to be naturally released from platelets in the bloodstream. In this study, polyP, which had been encapsulated by silica nanoparticles to prevent degradation of the active core, effectively disrupted binding between S proteins and ACE2 via specific amino acids in the S RBD. Binding assays showed that a concentration of 1 µg mL^−1^ of each ingredient (soluble polyP and polyP with silica/active ingredient nanoparticles), could both stop infection. This concentration is also close to the natural level of polyP in the bloodstream. Binding inhibition strength was also measured in varying environments including different temperatures, ionic strength, and pH.^[^ ^147^ ^]^

A polymeric composite in the form of a nasal spray is also proposed against SARS‐CoV‐2, reporting both prophylaxis and prevention. Moakes et al.^[^ ^148^ ^]^ reported a study using gellan and *λ*‐carrageenan in different ratios and developed a nasal spray reportedly blocking virus entrance into cells, inactivating the virus, and allowing removal of the virus from the upper respiratory system by the viscous medium owing to the mucoadhesive properties of the materials used. Authors suggested that results were promising to create a reliable device to stop infections before it passes through the nasal cavity and believed that the inhibition takes place in a three‐step mechanism, which is creating a steric barrier around the cell interface after applying the spray, which is followed by adsorption of the polymer to the virus and finally physically capturing the virus in the created mucoadhesive layer, respectively.^[^ ^148^ ^]^

**Figure 3:**
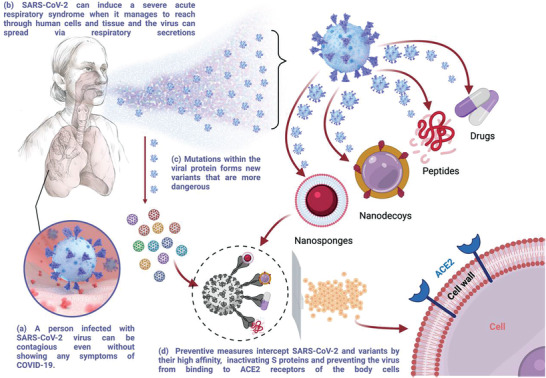
Schematic illustration of the SARS‐CoV‐2 transmission and scope of preventive measures discussed in this review. Created with BioRender.com.

In addition to drugs, nano‐decoys, synthetic peptides, and/or polymeric materials; natural materials have also been tested for their ability to interfere with S protein/ACE2 binding. A preprint by Liu et al.^[^ ^149^ ^]^ explored the use of epigallocatechin gallate, one of the main ingredients of green tea. In this study of SARS‐CoV‐2 and pseudoinverses of certain variants such as Alpha, Beta, and Epsilon, viral infection was prevented before the cell entry phase.^[^ ^149^ ^]^ It is noteworthy that the compound could block the affinity between S protein and ACE2 despite the key S mutations, which would be an important consideration as variants continue to evolve. Moreover, graphene and its derivatives have proved to be popular materials that are widely used against microorganisms owing to their antibacterial and antiviral characteristics.^[^ ^150^ ^]^ A recent paper reported that it was possible to modify the shape of the S protein and its RBDs by introducing heparin‐ and glycosaminoglycan‐based antivirals in which sulfates including versions of graphene oxide were also included. The concept was developed from previous studies of photothermal inactivation of herpes virus, in which sulfonated magnetic nanoparticles functionalized with reduced graphene oxide successfully captured near infrared light and destroyed the virus. Therefore, the authors proposed that the same mechanism could be applied to SARS‐CoV‐2, but more research is required on this topic.^[^ ^151^ ^]^ Finally, **Figure** **3** summarizes the preventive measures designed to interfere with SARS‐CoV‐2 S protein/ACE2 interactions.

### 4.2 Inanimate Object Approach

In addition to contributing to novel therapeutic approaches, materials science can also contribute to the development of new antiviral surfaces to prevent inanimate objects from becoming fomites that contribute to indirect viral transmission. Compared with antiviral vectors that are designed to bind viruses, the opposite effect is required: Materials and/or coatings that can repel SARS‐CoV‐2 and its variants and minimize how long they remain infectious. Considering certain characteristics of the virion, such as having its negatively charged genetic material enveloped by a positively charged capsid, SARS‐CoV‐2 was found to be more positively charged than its previous close relatives due to four more positively charged and five less negatively charged residues within.^[^ ^136^, ^152^ ^]^ Therefore, it would be wise to start with a positively charged surface to create electrostatic repulsion of positively charged SARS‐CoV‐2. Moreover, to optimize the efficiency of the surface, a polymeric phase could be used to create such surfaces, with the material selected from a broad range of available polymers to provide high porosity with low surface roughness, hydrophilicity, and low pH—all of which have been shown to repel viruses and minimize their window of infectivity.^[^ ^136^, ^153^ ^]^ In support of this idea, a recent study showed that positively charged chitosan created using the electrospraying method could create surfaces that repel viruses. Electrospraying is a reliable and well‐proven method to produce nano‐ and micro‐sized morphologies to create reliable, durable, and biologically compatible surfaces. The method is also very suitable for processing polymers and other materials to fabricate products for a broad range of applications.^[^ ^152^ ^]^ Moreover, the combination of electrospraying and electrospinning can be used to create positively‐charged fiber surfaces loaded with particles that could alter the surface further as desired and more recent processes like pressurized gyration with core‐sheath fibers can also be used to advantage here.^[^ ^154^, ^155^ ^]^ For example, such fibers could carry metallic nanoparticles at the surface only to shorten the activity window of viruses on the surface by incorporating copper into the polymeric matrix. There is a wide spectrum of metal‐based nanoparticles that have been used in a broad range of biomedical applications.^[^ ^156^ ^]^ Copper has already been identified as one of the best materials to counter SARS‐CoV‐2 on surfaces, inactivating the virus and reducing transmission and infectability rates.^[^ ^157^ ^]^ Furthermore, copper oxide has also been used to create coating materials to shorten virus activity time on surfaces. In the study reported by Behzadinasab et al.^[^ ^158^ ^]^ polyurethane—a popular polymer already widely used for coating—was used together with copper oxide to cover inanimate objects. Even though the study reported no virus‐repelling ability, 1 h was enough to inactivate the virus, providing 99.9% viral titer reduction on glass and stainless steel coated with this mixture.^[^ ^158^ ^]^ Furthermore, superhydrophobic coatings have also been studied to create SARS‐CoV‐2 repelling surfaces. In one study, a nanocomposite structure was specially designed to repel droplets carrying the virus, exploiting its high hydrophobic features. Using an ultrasonicator and acetone as a solvent, silica nanoparticles were incorporated with silicone or epoxy, and then polymerization was conducted. The researchers attempted to transfer these superhydrophobic properties to various surfaces by applying the material as coating or producing them in the shape of blocks, while the mechanical properties w also adjusted by altering the silicone/epoxy ratio. Results indicated that the surfaces were able to completely deflect droplets of approximately 10 *μ*L, and the rebound interval of the droplets was around 16 ms.^[^ ^159^ ^]^ It was also reported that the droplets generated by coughing, sneezing, or speaking can act as vectors to spread SARS‐CoV‐2, and droplet volumes of 1nl to 10nl correspond to the diameter of 125–270 µm. Hence, they are smaller than those tested and might rather be expected to have deflected more easily; however, further studies are needed.^[^ ^159^, ^160^ ^]^

## 5 Conclusions

Since the COVID‐19 pandemic began and progressed at an unbridled pace, scientists have made substantial progress in developing and rolling out successful vaccines. This advance is currently the most successful defensive countermeasure we have, as we still lack a definitive cure for COVID‐19. However, recent studies indicated SARS‐CoV‐2 is steadily mutating and giving rise to a new and diverse range of variants with improved transmissibility and infectivity. This evolutionary dynamic endangers current vaccination programs as the variants may eventually escape from immunity elicited by the first generation of inoculations. Further detailed understanding of the physical and chemical properties of the SARS‐CoV‐2 virus will aid efforts from the physical and materials sciences to develop novel ways to impair the ACE2 binding affinity of the virus and other properties that facilitate infection—regardless of which viral mutations arise. Research discussed in this review has revealed that using a materials science and engineering approach, promising antiviral therapeutic strategies can be developed by combining different materials from various categories such as, natural and synthetic materials, antiviral drugs, antibiotics and polymers, virus‐binding particles. In contrast, ex vivo approaches can reduce or eliminate viral persistence on the surfaces with which we interact daily; this parallel approach holds the promise of reducing the risk of indirect transmission. Hence, these methodologies reveal the extraordinary potential for developing ex vivo preventive measures, for safer environment and interactions that can intercept viruses before they attach to cells and prime the infection. However, further extensive research on the topic is crucial to develop preventive measures that can be effective regardless of the variant‐specific changes within the SARS‐CoV‐2 genome. Establishing greater collaboration and strong links between physical scientists, materials engineers, and clinicians is a vital requirement to extract the potential of current applications and develop new and more efficient ones in future.

## Conflict of Interest

The authors declare no conflict of interest.
